# Healthy lifestyle as a protective factor against the risk of gastroesophageal reflux disease: Prospective population cohort analysis

**DOI:** 10.1371/journal.pone.0360299

**Published:** 2026-10-15

**Authors:** Lei Peng, Xueqin Li, Jinfeng Qi, Liming Zhang, Zhenqing Yang, Xucheng Wu, Xueqing Jia, George O. Agogo, Guoju Liu, Kongxi Zhu, Zuyun Liu, Genxiang Mao, Honglei Wu

**Affiliations:** 1 Department of Gastroenterology, The Second Hospital, Cheeloo College of Medicine, Shandong University, Jinan, Shandong, China; 2 Center for Clinical Big Data and Analytics of the Second Affiliated Hospital, and Department of Big Data in Health Science School of Public Health, the Key Laboratory of Intelligent Preventive Medicine of Zhejiang Province, Zhejiang University School of Medicine, Hangzhou, Zhejiang, China; 3 Division of Global Health Protection, U.S. Centers for Disease Control and Prevention (CDC), Nairobi, Kenya; 4 Department of Physiology, Shandong Medical College, Jinan, Shandong, China; 5 Zhejiang Key Laboratory of Geriatrics and Geriatrics Institute of Zhejiang Province, Zhejiang Hospital, Hangzhou, China; Charite Universitatsmedizin Berlin, GERMANY

## Abstract

**Background:**

Lifestyle modifications are recommended by American Gastroenterological Association (AGA) for managing gastroesophageal reflux disease (GERD), a significant health issue; but evidence is scarce. This study aims to investigate the longitudinal association between healthy lifestyle behaviors and the risk of GERD.

**Methods:**

We analyzed data from 306,543 participants aged between 38 and 73 years without GERD at baseline from the UK Biobank. A summarized healthy lifestyle score (range, 0–7) was calculated using seven items: never smoking, healthy sleep, moderate body mass index (BMI), regular physical exercise, moderate alcohol intake, healthy diet, and moderate tea/coffee intake. Multivariable Cox regression was performed to examine the associations.

**Result:**

During a mean follow-up of 12.7 years, 27,943 (9.1%) GERD cases were identified. Each healthy lifestyle, except for moderate alcohol intake (hazard ratios [HR], 1.00; 95% confidence interval [CI]: 0.98–1.03) was associated with a lower risk of GERD. Relative to participants with less than 2 of summarized healthy lifestyle score, those with 2, 3, 4, and 5–7 of this score showed a significantly lower risk of GERD, with the HR of 0.94 (95% CI: 0.90–0.98), 0.83 (0.80–0.86), 0.74 (0.71–0.77), 0.63 (0.60–0.66), respectively (*P* for trend <0.001). Stratified analysis shows that the above associations were more pronounced among participants who were younger (< 60 years), female and employed (all *P* for interaction <0.05). Sensitivity analyses demonstrated similar results.

**Conclusions:**

This study highlights the importance of adherence to healthy lifestyle behaviors in reducing the prevalence of GERD in the general population.

## Introduction

Gastroesophageal reflux disease (GERD) is characterized by the presence of troublesome reflux or erosive complications in the esophagus due to the retrograde flow of refluxate into the esophagus [1], affecting approximately 13% of the global population [2,3]. The recognized sequelae of GERD include esophagitis, esophageal strictures, Barrett esophagus, and esophageal adenocarcinoma [2,3]. GERD has been a significant health and social issue that impairs quality of life and imposes substantial economic burdens [4]. The pathogenesis of GERD is complex and multifactorial, involving an interplay of chemical, mechanical, psychologic, and neurologic mechanisms. The clinical management of GERD is widely recognized to require a multifaceted approach, including lifestyle modifications and pharmaceutical interventions, as also reflected in the recommendations of the American Gastroenterological Association (AGA) [5]. However, given the limitations of proton pump inhibitors (PPIs) in addressing GERD symptoms and concerns over long-term side effects, there remains a lack of safer and more effective options for managing GERD [6]. In this context, increasing attention has been paid to lifestyle behaviors not only as part of symptom management but also as potential preventive factors for reducing GERD risk.Previous studies have demonstrated that some modifiable lifestyle behaviors including obesity [7,8], smoking [9–11], sleep [12,13], tea or coffee intake [14,15], and diet [7,16,17] are independently associated with GERD. However, the association between alcohol consumption and physical activity with GERD symptoms has yielded inconsistent findings in previous studies [9,18,19]. To date, longitudinal evidence between overall lifestyle behaviors and the risk of GERD is scarce, limiting the formation of preventive and intervention programs of GERD in public health.

Therefore, using data from a large population-based cohort, the UK Biobank (UKB), we aimed to assess the association of seven healthy lifestyle behaviors (never smoking, healthy diet, moderate BMI, healthy sleep, moderate alcohol intake, regular physical exercise, and moderate tea/coffee intake) with the risk of incident GERD.

## Methods

### Study population

UK Biobank is a large population-based cohort study with approximately 500,000 participants recruited across the United Kingdom between 2006 and 2010 [20]. Adults in the UKB were recruited within 6–12 months through 22 assessment centers across England, Scotland, and Wales. Data was collected through touchscreen questionnaires and a verbal interview on demographics, health status and lifestyle details as baseline assessments. The North West Multi-center Research Ethics Committee provided ethical approval for the UKB database. All participants have provided informed, written consent at recruitment. More details can be accessed on the official UKB website (https://www.ukbiobank.ac.uk/). Among 502,018 UK Biobank participants, those with prevalent GERD at baseline (n = 36,705) were excluded. Next, participants with missing data on any of the seven lifestyle behaviors (diet, n = 32,607; sleep, n = 32,135; regular physical activity, n = 6124; BMI, n = 1787; smoking, n = 5146; alcohol intake, n = 80,901; tea/coffee intake, n = 1148) were excluded. Finally, 306,543 participants were included (S1 Fig).

S1 Fig Flow chart illustrating selection of the study population in UK Biobank from 2006 to 2010.

### Ascertainment of exposure

In line with the literature, we considered seven healthy lifestyle behaviors (never smoking, healthy sleep, moderate BMI (18.5–24.9 kg/m^2^), regular physical exercise, moderate alcohol intake, healthy diet, and moderate tea/coffee intake) assessed at baseline in UKB [21].

According to the recommendations of the World Health Organization, never smoking was defined as smoking no more than 100 cigarettes in life [21]. The quality of sleep was determined based on self-reported sleep duration and sleep problems at baseline. Healthy sleep behaviors included five items: 1) ‘sleep duration 7 to 8 hours per day’ [22]; 2) ‘never or rarely experience sleeplessness/insomnia symptoms’ [23]; 3) ‘no self-reported snoring’ [24]; 4) ‘infrequent napping (‘never/rarely’ or ‘sometimes’)’ [25]; 5) ‘easy getting-up (‘fairly easy’ and ‘very easy’)’. Each item was assigned a score of 1 if the participant responded “yes”; otherwise, was assigned a score of 0. The cumulative sleep score was the sum of the single score and ranged from 0 to 5, with higher scores indicating a better level of sleep quality. Healthy sleep was defined as participants who had a cumulative sleep score of four points or higher [26]. Regular physical exercise was considered as healthy when it comprised either: 1) more than 150 minutes or 5 days per week of moderate physical activity; or 2) more than 75 minutes or once per week of vigorous activity [27]. Moderate alcohol intake was defined as no more than one alcoholic drink for females and two drinks for males, according to the dietary guidelines in the UK (one drink contains 8 g of ethanol in the UK) [21]. A healthy diet was defined as participants who met or surpassed recommended intake levels for over half of the following key food components: vegetables (3 servings/day), fruits (3 servings/day), whole grains (3 servings/day), (shell)fish (≥2 servings/week), vegetable oils (2 servings/day), refined grains (≤2 servings/day), processed meat (≤1 serving/week), unprocessed meat (≤2 servings/week), sugar-sweetened beverages (never) [28]. A healthy BMI was defined as a BMI with range of 18.5–24.9 kg/m^2^ [29]. Moderate tea or coffee intake were defined as no more than two cups per day [14].

Each healthy lifestyle behavior was assigned a score of 1, and otherwise 0. The participants with a summarized score of 0 (n = 5043), 1 (n = 27,217), and 5 (n = 41,869), 6 (n = 14,368), and 7 (n = 1972) were pooled together as a single group to increase the sample size for analysis, respectively. Thus, based on their summarized scores, participants were categorized into five groups: scores of 0–1, 2, 3, 4, and 5–7.

### Ascertainment of outcome

The incident GERD was recorded using the International Classification of Diseases 10th revision code (ICD-10) and defined as code K21 for GERD(UK Biobank Field ID 131585 for diagnosis and 131584 for diagnosis date). The UKB has established a comprehensive category that documents the initial occurrence dates of a range of health-related outcomes from several sources, including primary care data, hospital admissions data, self-reported medical conditions, and death register records. The health-related outcomes were mapped to ICD-10 and updated regularly, and were used to identify GERD. The time-to-event was calculated from the date of recruitment (between 2006 and 2010) to the date of the first diagnosis of GERD, date of death, date of loss to follow-up, or end of follow-up, whichever came first.

### Ascertainment of covariates

Covariates included age (<60 years, ≥ 60 years), gender (female, male), ethnics (white, non-white), Townsend deprivation index (TDI), occupation, education, history of cancer (Yes, No), diabetes (Yes, No), hypertension (Yes, No), depression (Yes, No), anxiety (Yes, No), and upper gastroenterology diseases (UGID, Yes or No). Details are given in (S1 Table).

### Statistical analysis

The baseline characteristics of the study participants were described according to the number of healthy lifestyle behaviors. Continuous variables were presented as the mean (standard deviation) and categorical variables were presented as counts (percentages). Continuous variables were compared by analysis of variance, and categorical variables were compared by Chi-Squared test. Cox proportional hazard regression models were applied to estimate the associations of healthy lifestyle behaviors with the risk of GERD. We computed multivariable hazard ratios (HRs) and corresponding 95% confidence intervals (CIs) from two models. Model 1 adjusted for age and gender. Model 2 additionally adjusted for TDI, ethnics, occupation, education, history of cancer, diabetes, hypertension, depression, anxiety, and UGID. The proportional hazards assumption of the models was tested using Schoenfeld residuals, and no significant deviation from the assumption was found.

Subgroup analyses were conducted by age (<60 years or ≥60 years), gender (male or female), ethnics (white or non-white), education level (high, intermediate, or low), and occupation (employed or unemployed). Moreover, we explored potential effect modifications by assessing interactions between healthy lifestyle behaviors and each stratifying variable.

Several sensitivity analyses were conducted to test the robustness of our results. First, we repeated the main analysis after excluding participants taking anti-acid drugs at baseline such as PPIs, Histamine 2 Receptor antagonists (H_2_RAs) (The identification codes are detailed in S2 Table). Second, we repeated the main analysis after excluding participants with cancer, diabetes, hypertension, anxiety, depression, and UGID at baseline, respectively. Third, the healthy lifestyles score was subsequently categorized as favorable (score: 5–7), intermediate (score: 3–4), and unfavorable (score: 0–2) lifestyles, and then we repeated the main analyses. Fourth, we performed multiple imputation on the missing data for lifestyle behaviors. Fifth, to reduce the potential reverse causation, we repeated the main analyses after excluding outcomes that occurred within the first 2 years of follow-up.

A two-sided P value <0.05 was considered to be statistically significant. All analyses were performed using R software (version 4.2.1), R Studio and R packages were utilized: (survival, ggplot2, dplyr etc).

## Results

### Baseline characteristic

The mean age of the 306, 543 participants was 56.9 years. Among all participants, 32,260 (10.5%) had less than 2 of the 7 healthy lifestyle behaviors, 61,299 (20.0%) had 2 healthy behaviors, 81,963 (26.7%) had 3 healthy behaviors, 72,812 (23.8%) had 4 healthy behaviors, 58,209 (19.0%) had 5–7 healthy behaviors. Compared with participants who had no more than 2 healthy behaviors, those who adhered to 5–7 healthy behaviors were more likely to be younger, female, employed, have lower TDI, and with high level of education. The detailed baseline characteristics of the study participants are shown in Table 1. The *P* values were generated using χ2 and analysis of variance for categorical and continuous variables, respectively.

**Table 1:** Baseline characteristics of the study participants in total and according to the No. of healthy lifestyle behaviors.

No. of healthy lifestyle behaviors^a^							
	Overall	0-1	2	3	4	5-7	
Characteristics	n = 306,543	n = 32,260	n = 61,299	n = 81,963	n = 72,812	n = 58,209	*P* value^b^
**Age (years)**							<0.001
<60	164,047 (53.5)	16,408 (50.9)	31,322 (51.1)	43,100 (52.6)	39,931 (54.8)	33,286 (57.2)	
≥60	142,496 (46.5)	15,852 (49.1)	29,977 (48.9)	38,863 (47.4)	32,881 (45.2)	24,923 (42.8)	
**Gender**							<0.001
Female	154,652 (50.5)	12,883 (39.9)	26,702 (43.6)	39,969 (48.8)	39,719 (54.6)	35,379 (60.8)	
Male	151,891 (49.5)	19,377 (60.1)	34,597 (56.4)	41,994 (51.2)	33,093 (45.4)	22,830 (39.2)	
**Ethnics**							<0.001
White	295,654 (96.7)	31,573 (98.1)	59,585 (97.5)	79,186 (96.8)	69,755 (96.0)	55,555 (95.7)	
Non-White	10,101 (3.3)	599 (1.9)	1533 (2.5)	2577 (3.2)	2877 (4.0)	2515 (4.3)	
**Occupation**							<0.001
Employed	288,097 (94.2)	29,402 (91.3)	57,186 (93.5)	77,315 (94.5)	68,934 (94.9)	55,260 (95.2)	
Unemployed	17,735 (5.8)	2786 (8.7)	3968 (6.5)	4463 (5.5)	3702 (5.1)	2816 (4.8)	
**TDI** (mean (SD))	−1.6 (2.9)	−1.2 (3.1)	−1.4 (3.0)	−1.6 (2.9)	−1.8 (2.8)	−1.9 (2.7)	<0.001
**Education** ^**c**^							<0.001
High	111,241 (36.5)	8628 (26.9)	18,788 (30.9)	28,283 (34.7)	28,822 (39.8)	26,720 (46.1)	
Intermediate	100,612 (33.0)	10,940 (34.1)	20,599 (33.8)	27,116 (33.3)	23,604 (32.6)	18,353 (31.7)	
Low	92,912 (30.5)	12,503 (39.0)	21,523 (35.3)	26,068 (32.0)	19,962 (27.6)	12,856 (22.2)	
**Cancer**							0.017
No	281,189 (91.7)	29,519 (91.5)	56,121 (91.5)	75,146 (91.7)	66,836 (91.8)	53,567 (92.0)	
Yes	25,354 (8.3)	2741 (8.5)	5178 (8.5)	6817 (8.3)	5976 (8.2)	4642 (8.0)	
**Diabetes**							<0.001
No	292,213 (95.3)	29,904 (92.7)	57,614 (94.0)	77,948 (95.1)	70,086 (96.3)	56,661 (97.3)	
Yes	14,330 (4.7)	2356 (7.3)	3685 (6.0)	4015 (4.9)	2726 (3.7)	1548 (2.7)	
**Hypertension**							<0.001
No	139,162 (45.4)	10,892 (33.8)	23,687 (38.6)	35,612 (43.5)	35,908 (49.3)	33,063 (56.8)	
Yes	167,381 (54.6)	21,368 (66.2)	37,612 (61.4)	46,351 (56.5)	36,904 (50.7)	25,146 (43.2)	
**Depression**							<0.001
No	291,635 (95.1)	29,865 (92.6)	57,584 (93.9)	77,860 (95.0)	69,892 (96.0)	56,434 (97.0)	
Yes	14,908 (4.9)	2395 (7.4)	3715 (6.1)	4103 (5.0)	2920 (4.0)	1775 (3.0)	
**Anxiety**							<0.001
No	302,146 (98.6)	31,624 (98.0)	60,309 (98.4)	80,756 (98.5)	71,870 (98.7)	57,587 (98.9)	
Yes	4397 (1.4)	636 (2.0)	990 (1.6)	1207 (1.5)	942 (1.3)	622 (1.1)	
**UGID**							<0.001
No	303,874 (99.1)	31,831 (98.7)	60,613 (98.9)	81,210 (99.1)	72,308 (99.3)	57,912 (99.5)	
Yes	2669 (0.9)	429 (1.3)	686 (1.1)	753 (0.9)	504 (0.7)	297 (0.5)	
**Lifestyle behaviors**							
**Moderate BMI**							<0.001
No	203,873 (66.5)	30,834 (95.6)	52,864 (86.2)	61,189 (74.7)	42,079 (57.8)	16,907 (29.0)	
Yes	102,670 (33.5)	1426 (4.4)	8435 (13.8)	20,774 (25.3)	30,733 (42.2)	41,302 (71.0)	
**Moderate alcohol intake**							<0.001
No	112,123 (36.6)	26,373 (81.8)	33,871 (55.3)	29,803 (36.4)	16,330 (22.4)	5746 (9.9)	
Yes	194,420 (63.4)	5887 (18.2)	27 428 (44.7)	52,160 (63.6)	56,482 (77.6)	52,463 (90.1)	
**Never smoking**							<0.001
No	141,707 (46.2)	29,255 (90.7)	42,928 (70.0)	40,173 (49.0)	21,949 (30.1)	7402 (12.7)	
Yes	164,836 (53.8)	3005 (9.3)	18,371 (30.0)	41,790 (51.0)	50,863 (69.9)	50,807 (87.3)	
**Regular exercise**							<0.001
No	74,714 (24.4)	20,610 (63.9)	22,440 (36.6)	18,708 (22.8)	9772 (13.4)	3184 (5.5)	
Yes	231,829 (75.6)	11,650 (36.1)	38 859 (63.4)	63,255 (77.2)	63,040 (86.6)	55,025 (94.5)	
**Healthy diet**							<0.001
No	191,480 (62.5)	30,403 (94.2)	50,103 (81.7)	55,544 (67.8)	38,249 (52.5)	17,181 (29.5)	
Yes	115,063 (37.5)	1857 (5.8)	11,196 (18.3)	26,419 (32.2)	34,563 (47.5)	41,028 (70.5)	
**Healthy sleep**							<0.001
No	180,591 (58.9)	30,121 (93.4)	48,899 (79.7)	53,107 (64.8)	34,358 (47.2)	14,106 (24.2)	
Yes	125,952 (41.1)	2139 (6.6)	12,400 (20.3)	28,856 (35.2)	38,454 (52.8)	44,103 (75.8)	
**Moderate tea/coffee intake**							<0.001
No	245,004 (79.9)	31,007 (96.1)	55,390 (90.4)	69,328 (84.6)	55,699 (76.5)	33,580 (57.7)	
Yes	61,539 (20.1)	1253 (3.9)	5909 (9.6)	12,635 (15.4)	17,113 (23.5)	24,629 (42.3)	

a Healthy lifestyle behaviors included never smoking, moderate BMI(≥18.5 and <24.9 kg/m^2^), healthy sleep (at least four out of the following five items:1) sleep duration 7–8 hours per day; 2) never or rarely experience sleeplessness/insomnia symptoms; 3) no self-reported snoring; 4) infrequent napping (never/rarely or sometime); 5) easy getting-up (fairly easy or very easy)), regular physical activity (included:1) more than 150 minutes or 5 days per week of moderate physical activity; or 2) more than 75 minutes or once per week of vigorous activity)), moderate alcohol intake(no more than one alcoholic drink for females and two drinks for males), healthy diet (met or exceeded the recommended intake levels for over half of the following key food components: vegetables (3 servings/day), fruits (3 servings/day), whole grains (3 servings/day), (shell)fish (≥2 servings/week), vegetable oils (2 servings/day), refine grains (≤2 servings/day), processed meat (≤1 servings/week), unprocessed meat (≤2 servings/week), sugar-sweetened beverages(never)), and moderate tea/coffee intake (no more than two cups per day).

b The *P* values were generated using χ2 and analysis of variance for categorical and continuous variables, respectively.

c High educational level: college or university degree; Intermediate educational level: A/AS levels or equivalent, O levels/GCSEs or equivalent; Low educational level: none of the aforementioned. TDI, Townsend deprivation index; UGID, upper gastroenterology diseases; BMI, body mass index.

### The association between healthy lifestyle behaviors and the risk of GERD

A total of 27,943 (9.11%) participants developed GERD during a mean follow-up of 12.7 years. Fig 1 presents the percentages of incident GERD by each and summarized healthy lifestyle behaviors. Each healthy lifestyle, except moderate alcohol intake (10.02% versus 10.03%, *P* = 0.98) was associated with a lower percentage of incident GERD (All *P* < 0.03). Fig 2 displays the associations between healthy lifestyle behaviors and the risk of GERD. After adjusting for all covariates (in Model 2), healthier lifestyles behaviors were significantly associated with lower risk of GERD (*P* for trend <0.001). For instance, compared with participants having no more than 2 healthy behaviors, those having healthy behaviors of 2, 3, 4, 5–7 had decreased risks of GERD, with HRs of 0.94 (95% CI: 0.90–0.98), 0.83 (95% CI: 0.80–0.86), 0.74 (95% CI: 0.71–0.77), 0.63 (95% CI: 0.60–0.66), respectively. Except for moderate alcohol intake, moderate BMI, healthy sleep, never smoking, regular physical exercise, healthy diet, and moderate tea/coffee intake were associated with a decreased risk of GERD, with HRs of 0.75 (95% CI: 0.73–0.77), 0.74 (95% CI: 0.72–0.76), 0.86 (95% CI: 0.84–0.88), 0.86 (95% CI: 0.84–0.89), 0.93 (95% CI: 0.91–0.96), 0.93 (95% CI: 0.90–0.96), respectively.

**Fig 1:**
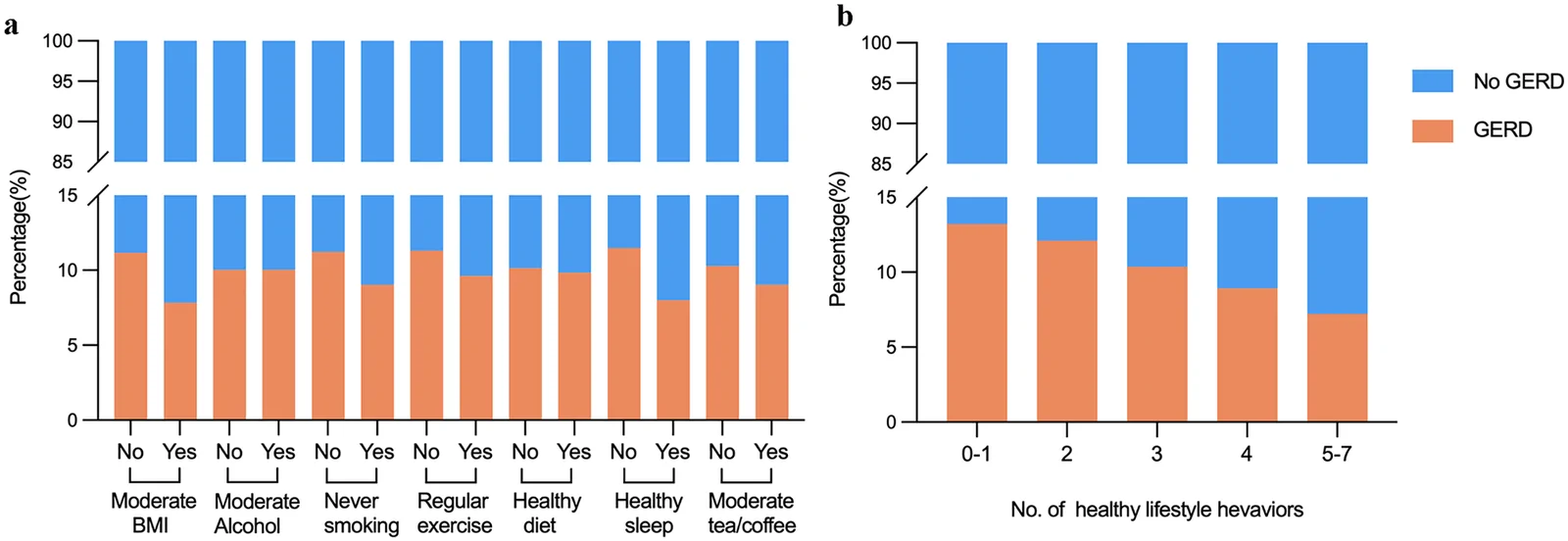
Incidence of gastroesophageal reflux disease according to individual and combined healthy lifestyle behaviors.

**Fig 2:**
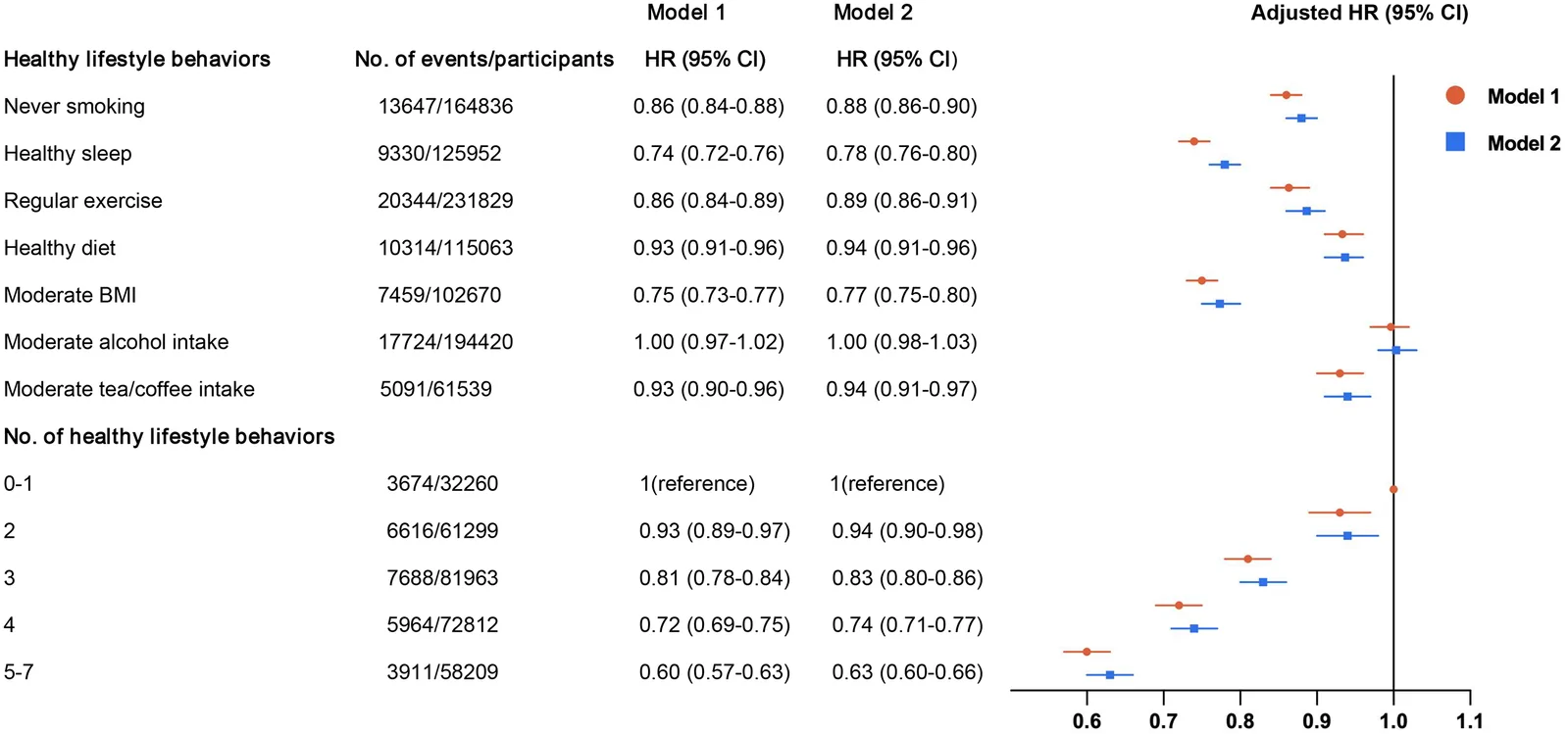
Multivariable-adjusted associations between healthy lifestyle behaviors and the risk of gastroesophageal reflux disease.

### Subgroup and sensitivity analyses

Table 2 presents the association between healthy lifestyle behaviors and the risk of GERD stratified by age, gender, ethnics, education, and occupation. The associations of healthy lifestyle behaviors were more pronounced among younger (< 60 years) (*P* for interaction <0.001), female (*P* for interaction = 0.001), and employed participants (*P* for interaction = 0.002). Among participants aged below 60 years, compared with participants having no more than 2 healthy behaviors, those with healthy behaviors of 2, 3, 4, 5–7 had decreased risks of GERD, with HRs of 0.91 (95% CI: 0.86–0.97), 0.80 (95% CI: 0.76–0.85), 0.70 (95% CI: 0.66–0.74), 0.58 (95% CI: 0.55–0.63), respectively (*P* for trend <0.001).

**Table 2:** Association between healthy lifestyle behaviors and the risk of GERD stratified by subgroups.

		HR (95% CI)^a^						
		Number of healthy lifestyle behaviors^b^						
Subgroups	No. of events/ participants	0-1	2	3	4	5-7	*P* for trend^c^	*P* for interaction
**Age groups(years)**								<0.001
<60	12,474/164,047	1(reference)	0.91 (0.86-0.97)	0.80 (0.76-0.85)	0.70 (0.66-0.74)	0.58 (0.55-0.63)	<0.001	
≥60	15,469/14,2496	1(reference)	0.97 (0.91-1.02)	0.85 (0.81-0.90)	0.78 (0.73-0.82)	0.67 (0.63-0.71)	<0.001	
**Gender**								0.001
Female	14,367/154,652	1(reference)	0.94 (0.88-0.99)	0.80 (0.76-0.85)	0.71 (0.67-0.75)	0.61 (0.57-0.65)	<0.001	
Male	13,576/151,891	1(reference)	0.94 (0.89-0.99)	0.85 (0.81-0.90)	0.77 (0.73-0.81)	0.65 (0.61-0.70)	<0.001	
**Ethnics**								0.260
White	27,074/295,654	1(reference)	0.93 (0.90-0.97)	0.81 (0.78-0.85)	0.71 (0.68-0.74)	0.59 (0.56-0.62)	<0.001	
Non-white	807/10,101	1(reference)	0.87 (0.46-1.65)	0.75 (0.40-1.41)	0.70 (0.37-1.33)	0.67 (0.34-1.33)	0.005	
**Education**								0.900
High	7728/111,241	1(reference)	0.97 (0.89-1.05)	0.81 (0.74-0.88)	0.74 (0.68-0.80)	0.64 (0.59-0.70)	<0.001	
Intermediate	8976/100,612	1(reference)	0.93 (0.87-1.00)	0.80 (0.74-0.86)	0.72 (0.67-0.78)	0.61 (0.56-0.66)	<0.001	
Low	11,041/92,912	1(reference)	0.93 (0.88-0.99)	0.87 (0.82-0.92)	0.75 (0.71-0.80)	0.63 (0.59-0.68)	<0.001	
**Occupation**								0.002
Employed	25,916/288,097	1(reference)	0.91 (0.80-1.03)	0.74 (0.65-0.85)	0.61 (0.52-0.71)	0.55 (0.46-0.66)	<0.001	
Unemployed	1941/17,735	1(reference)	0.95 (0.91-0.99)	0.84 (0.80-0.87)	0.75 (0.72-0.79)	0.64 (0.61-0.67)	<0.001	

a The Cox regression model was adjusted for age, gender, Townsend deprivation index, ethnics, occupation, education, history of cancer, diabetes, hypertension, depression, anxiety, and upper gastroenterology disease. In each stratified analysis, the stratification variable was excluded in the model.

b Healthy lifestyle behaviors included never smoking, moderate BMI(≥18.5 and <24.9 kg/m^2^), healthy sleep (at least four out of the following five items:1) sleep duration 7–8 hours per day; 2) never or rarely experience sleeplessness/insomnia symptoms; 3) no self-reported snoring; 4) infrequent napping (never/rarely or sometime); 5) easy getting-up (fairly easy or very easy)), regular physical activity (included:1) more than 150 minutes or 5 days per week of moderate physical activity; or 2) more than 75 minutes or once per week of vigorous activity)), moderate alcohol intake(no more than one alcoholic drink for females and two drinks for males), healthy diet (met or exceeded the recommended intake levels for over half of the following key food components: vegetables (3 servings/day), fruits (3 servings/day), whole grains (3 servings/day), (shell)fish (≥2 servings/week), vegetable oils (2 servings/day), refine grains (≤2 servings/day), processed meat (≤1 servings/week), unprocessed meat (≤2 servings/week), sugar-sweetened beverages(never)), and moderate tea/coffee intake (no more than two cups per day).

GERD, gastroesophageal reflux disease; HR, hazard ratio; CI, confidence interval.

^c^Calculated to test linear trend using healthy lifestyle behaviors (5 categories) as a continuous variable.

GERD, gastroesophageal reflux disease; HR, hazard ratio; CI, confidence interval.

Sensitivity analyses suggested similar associations when 1) excluding participants with oral PPI/H2RA or anti-acid therapy on the onset of GERD (S1 Table); 2) excluding participants with anxiety, depression, diabetes, hypertension, cancer at baseline (S2-S6 Tables); 3) excluding participants diagnosed with other UGIDs (esophagitis, Barrett’s esophagus, esophageal stricture, esophageal varicies, gastric/stomach ulcers, gastritis/gastric erosions, gastrointestinal bleeding) (S7 Table); 4) subsequently categorizing the healthy lifestyles behaviors as favorable (healthy lifestyle score: 5–7), intermediate (healthy lifestyle score: 3–4), and unfavorable (healthy lifestyle score: 0–2) lifestyles (S8 Table); 5) performing multiple imputation on the missing data of lifestyle behaviors (S9 Table); and 6) excluding participants with the onset of GERD within 2 years of follow-up (S10-S11 Tables).

## Discussion

This study demonstrated that adherence to a variety of healthy lifestyle behaviors—including moderate BMI, never smoking, healthy sleep, healthy diet, moderate alcohol intake, regular physical exercise, and moderate tea/coffee intake—was strongly linked that subjects who report healthy lifestyle behaviors have a lower risk of GERD later. to a lower risk of GERD. Further subgroup analysis revealed that adherence to these healthy lifestyles was particularly pronounced in lowering the risk of GERD among middle-aged participants, female, and those who are currently employed. To our knowledge, this is the largest population-based longitudinal study to demonstrate that adherence to a healthier lifestyle reduced the risk of GERD among the middle-aged and older adults.

According to American College of Gastroenterology (ACG) Clinical Guideline, PPIs remain the first choice for the treatment of GERD [5]. However, up to 50% of patients fail to find sufficient relief with empirical PPIs therapy [6], and there is mounting concern over long-term safety and over-prescription due to numerous studies questioning adverse effects [2,30–34]. It is imperative to shift our focus towards exploring alternative preventive or therapeutic measures standard medication. Our study emphasizes the critical significance of summarized healthy lifestyle behaviors in the primary prevention of GERD. A recent cross-sectional analysis of 3363 Iranian adults reported an inverse relationship between a comprehensive healthy lifestyle and the prevalence of GERD [35]. They calculated the “healthy lifestyle score” for each participant with five lifestyle factors, including dietary habits, dietary intakes, psychological distress, smoking and physical activity. However, this study found that there was no association between individual lifestyle factor, such as smoking, physical activity, healthy dietary habits, and GERD. In contrast, our study revealed a relationship between smoking, physical activity, and the risk of GERD. Another cohort study based on the Nurses’ Health Study II showed that adherence to antireflux lifestyle factors (including normal weight, never smoking, moderate-to-vigorous physical activity, prudent diet and no more than 2 cups of coffee, tea, or soda daily) can significantly decrease the incidence of GERD [14]. Building on this above study, we extended the findings to 151,891 men and emphasized the crucial role of healthy sleep in the risk of GERD.

The lower risk of GERD for those who never smoked could be explained by the fact that cigarette smoking prolonged acid clearance and a decrease in the pressure of the lower esophageal sphincter [36,37], as well as increased frequency of reflux episodes [38]. A population-based study in Sweden among 820 adults showed no association between BMI and the severity or duration of reflux symptoms [39], and this finding is inconsistent with ours that showed obesity as a risk factor of GERD. But it is widely accepted that weight loss can positively influence reflux symptoms in obese individuals. Consistent with previous studies [7,40], we suggest that diet is the major aggravating factor of GERD, possibly because it stimulates the production of gastric acid available for reflux into the esophagus. Besides, a variety of specific food have been identified as potentially aggravating reflux in GERD patients such as raw onions [41], chocolate [42], peppermint [43], citrus juices [44], and spicy food [45,46].

Strenuous exercise [47,48], especially vigorous exercise [49–51], can lead to an excessive occurrence of reflux in individuals with GERD. However, the influence of exercise at low or moderate intensity remains uncertain. Our study revealed that adherence to regular physical exercise can reduce the risk of GERD. Thus, while we affirm the benefits of exercise for GERD prevention, more research is required to determine the optimal types and intensities of exercise to reduce GERD risks. A bidirectional relationship exists between GERD and sleep, with GERD often causing various sleep disturbances [52,53], and improvements in sleep having a positive influence on GERD [54–56]. Antireflux treatments have proven effective in alleviating sleep disturbances and enhancing sleep quality [57–59]. However, few longitudinal studies have explored this association regarding the onset of GERD. Our present study confirms that healthy sleep significantly reduces the risk of GERD. This highlights the importance of monitoring sleep disturbances in GERD patients and emphasizes the need for GERD prevention strategies targeting individuals with sleep disorders, as they may be at heightened risk. Despite inconsistent or weak evidence linking tea and coffee consumption to GERD risk, and lack of solid clinical evidence for their removal to improve symptoms, current guidelines do not typically advocate reducing or eliminating these beverages for GERD management [5]. A study based on Nurses’ Health Study II suggested that restricting the daily intake of coffee, tea, or soda to two cups or less could lower the risk of GERD symptoms. In line with prior research, our findings echo that consistently keeping tea and coffee consumption under two cups per day notably reduces the risk of GERD.

Our study revealed that moderate alcohol intake does not reduce the risk of GERD corroborating previous findings from an Mendelian randomization study [60]. However, despite inconclusive findings, we endorse the general advice to consume alcohol moderately as part of overall health risk reduction strategies, including GERD-related risks. Although moderate alcohol intake alone didn’t show an association to GERD prevention among the seven assessed healthy lifestyle behaviors, the combined effect of all seven behaviors proved notably beneficial. Therefore, adherence to all seven health-promoting behaviors remains prudent, considering their broader health benefits extending beyond just GERD prevention.

The present study demonstrated notable strengths, including a large sample size, a prospective design, and an extensive follow-up duration. Additionally, we have considered a variety of lifestyle behaviors that can provide actionable implications for prevention efforts. Public health programs could integrate these lifestyle factors into population-level education to mitigate GERD burden. Clinicians may also incorporate these insights into routine counseling by promoting healthy sleep, weight management, and balanced behavioral habits as evidence-supported strategies for reducing GERD risk. This study also has a few limitations. First, some GERD patients may present with atypical symptoms such as hoarseness, cough, throat clearing, and sore throat, which could lead to underdiagnosis of GERD [5,61]. Second, GERD cases in our study were identified using ICD-10 codes and UK Biobank outcome fields, which may preferentially capture clinically recognized or more severe cases, while missing mild, self-managed, atypical, or functional reflux-related symptoms, potentially introducing outcome misclassification. If this underdiagnosis is non-differential across lifestyle groups, our effect estimates would likely be biased toward the null, suggesting that the true protective associations may be stronger than observed. Conversely, differential underdiagnosis (e.g., healthier individuals being more likely to seek care or less likely to report mild symptoms) would make the direction of bias less predictable. Nonetheless, the prospective design reduces recall and selection biases, and the use of validated health records ensures high specificity for clinically significant GERD cases. Future studies incorporating symptom-based screening or ambulatory pH monitoring are warranted to capture the full GERD spectrum and further validate our findings. Additionaly, our longitudinal associations do not imply causality, hence more research is needed to confirm our findings. Third, we were unable to explore the different types and severity of GERD due to the sources of data and further classification is not consistent. Finally, the majority of UKB participants were White, lived at a higher socioeconomic level, and relatively healthy, which may limit the generalizability of our results to other ethnic and socioeconomic groups [62].

## Conclusions

This study demonstrates a significant association between adherence to multiple healthy lifestyle behaviors and a decreased risk of GERD, highlight the potential of adhering multiple healthy lifestyle behaviors in GERD management. Based on large-scale, long-term follow-up study, this findings provide solid evidence for the AGA’s approach to GERD management, thereby reinforcing the foundation upon which clinical guidelines and recommendations can be built.

## Supporting information

S1 Checklist Human subjects research checklist. (DOCX)

S1 Fig Flow chart of the study participants. GERD, gastroesophageal reflux disease; BMI, body mass index.
(DOCX)

S1 Table Associations between healthy lifestyle behaviors and risk of GERD (excluding participants with PPI/H2RA/anti-acid treatment at baseline, N = 287,893). Notes: GERD, gastroesophageal reflux disease; HR, hazard ratio; CI, confidence interval; BMI, body mass index; PPI, proton pump inhibitor; H2RA, Histamine-2 Receptor Antagonists. Model 1: adjusted for age, gender. Model 2: adjusted for age, gender, Townsend deprivation index, ethnics, occupation, education, history of cancer, diabetes, hypertension, depression, anxiety, upper gastroenterology diseases.
(DOCX)

S2 Table Associations between healthy lifestyle behaviors and risk of GERD (excluding participants with anxiety at baseline, N = 302,164). Notes: GERD, gastroesophageal reflux disease; HR, hazard ratio; CI, confidence interval; BMI, body mass index. Model 1: adjusted for age, gender. Model 2: adjusted for age, gender, Townsend deprivation index, ethnics, occupation, education, history of cancer, diabetes, hypertension, depression, upper gastroenterology diseases.
(DOCX)

S3 Table Associations between healthy lifestyle behaviors and risk of GERD (excluding participants with depression at baseline, N = 291,635). Notes: GERD, gastroesophageal reflux disease; HR, hazard ratio; CI, confidence interval; BMI, body mass index. Model 1: adjusted for age, gender. Model 2: adjusted for age, gender, Townsend deprivation index, ethnics, occupation, education, history of cancer, diabetes, hypertension, anxiety, upper gastroenterology diseases.
(DOCX)

S4 Table Associations between healthy lifestyle behaviors and risk of GERD (excluding participants with diabetes at baseline, N = 292,213). Notes: GERD, gastroesophageal reflux disease; HR, hazard ratio; CI, confidence interval; BMI, body mass index. Model 1: adjusted for age, gender. Model 2: adjusted for age, gender, Townsend deprivation index, ethnics, occupation, education, history of cancer, hypertension, depression, anxiety, upper gastroenterology diseases.
(DOCX)

S5 Table Associations between healthy lifestyle behaviors and risk of GERD (excluding participants with hypertension at baseline, N = 139,162). Notes: GERD, gastroesophageal reflux disease; HR, hazard ratio; CI, confidence interval; BMI, body mass index. Model 1: adjusted for age, gender. Model 2: adjusted for age, gender, Townsend deprivation index, ethnics, occupation, education, history of cancer, diabetes, depression, anxiety, upper gastroenterology diseases.
(DOCX)

S6 Table Associations between healthy lifestyle behaviors and risk of GERD (excluding participants with cancer at baseline, N = 281,189). Notes: GERD, gastroesophageal reflux disease; HR, hazard ratio; CI, confidence interval; BMI, body mass index. Model 1: adjusted for age, gender. Model 2: adjusted for age, gender, Townsend deprivation index, ethnics, occupation, education, history of diabetes, hypertension, depression, anxiety, upper gastroenterology diseases.
(DOCX)

S7 Table Associations between healthy lifestyle behaviors and risk of GERD (excluding participants with UGID at baseline, N = 291,635). Notes: GERD, gastroesophageal reflux disease; HR, hazard ratio; CI, confidence interval; BMI, body mass index. Model 1: adjusted for age, gender. Model 2: adjusted for age, gender, Townsend deprivation index, ethnics, occupation, education, history of cancer, diabetes, hypertension, depression, anxiety.
(DOCX)

S8 Table Associations between healthy lifestyle behaviors (favorable, intermediate, and unfavorable classification) and risk of GERD. Notes: GERD, gastroesophageal reflux disease; HR, hazard ratio; CI, confidence interval; BMI, body mass index. Healthy lifestyle behaviors were categorized as favorable (score: 5–7), intermediate (score: 3–4), and unfavorable (score: 0–2). Model 1: adjusted for age, gender. Model 2: adjusted for age, gender, Townsend deprivation index, ethnics, occupation, education, history of cancer, diabetes, hypertension, depression, anxiety, upper gastroenterology diseases.
(DOCX)

S9 Table Associations between healthy lifestyle behaviors and risk of GERD after multiple imputation on the missing data of lifestyle behaviors. Notes: GERD, gastroesophageal reflux disease; HR, hazard ratio; CI, confidence interval; BMI, body mass index. Model 1: adjusted for age, gender. Model 2: adjusted for age, gender, Townsend deprivation index, ethnics, occupation, education, history of cancer, diabetes, hypertension, depression, anxiety, upper gastroenterology diseases.
(DOCX)

S10 Table Associations between healthy lifestyle behaviors and risk of GERD (excluding participants onset of GERD within 2 years follow up, N = 302,564). Notes: GERD, gastroesophageal reflux disease; HR, hazard ratio; CI, confidence interval; BMI, body mass index. Model 1: adjusted for age, gender. Model 2: adjusted for age, gender, Townsend deprivation index, ethnics, occupation, education, history of cancer, diabetes, hypertension, depression, anxiety, upper gastroenterology diseases.
(DOCX)

S11 Table UK Biobank Field IDs of variables. (DOCX)

## Abbreviations

ACG: American College of Gastroenterology
AGA: American gastroenterological association
BMI: body mass index
CI: confidence interval
GERD: gastroesophageal reflux disease
H_2_RAs: Histamine 2 Receptor antagonists
HR: hazard ratios
ICD-10: international classification of diseases 10th revision code
PPI: proton pump inhibitors
UGID: upper gastroenterology diseases
UKB: UK Biobank
