# Supplementary material for: Healthy lifestyle as a protective factor against the risk of gastroesophageal reflux disease: Prospective population cohort analysis
Source: PLoS One. 2026 Oct 15;21(10):e0360299. doi: 10.1371/journal.pone.0360299 (PMC13671543; doi:10.1371/journal.pone.0360299)
Supplement: Supplementary file 2 [file pone.0360299.s002.docx]

**Figure S1. Flow chart of the study participants.** GERD, gastroesophageal reflux disease; BMI, body mass index.
