# Supplementary material for: Healthy lifestyle as a protective factor against the risk of gastroesophageal reflux disease: Prospective population cohort analysis
Source: PLoS One. 2026 Oct 15;21(10):e0360299. doi: 10.1371/journal.pone.0360299 (PMC13671543; doi:10.1371/journal.pone.0360299)
Supplement: Supplementary file 4 [file pone.0360299.s004.docx]

**Table S2. Associations between healthy lifestyle behaviors and risk of GERD (excluding participants with anxiety at baseline, N=302,164).**

Notes: GERD, gastroesophageal reflux disease; HR, hazard ratio; CI, confidence interval; BMI, body mass index.

Model 1: adjusted for age, gender.

Model 2: adjusted for age, gender, Townsend deprivation index, ethnics, occupation, education, history of cancer, diabetes, hypertension, depression, upper gastroenterology diseases.

|  | **Model 1** | |  | **Model 2** | |
| --- | --- | --- | --- | --- | --- |
| **Healthy lifestyle behaviors** | **HR (95% CI)** | ***P* value** |  | **HR (95% CI)** | ***P* value** |
| Moderate BMI | 0.72 (0.70-0.74) | <0.001 |  | 0.76 (0.74-0.78) | <0.001 |
| Moderate alcohol | 0.99 (0.97-1.02) | 0.487 |  | 1.01 (0.98-1.03) | 0.608 |
| Never smoking | 0.83 (0.81-0.85) | <0.001 |  | 0.87 (0.85-0.89) | <0.001 |
| Regular exercise | 0.86 (0.83-0.88) | <0.001 |  | 0.88 (0.85-0.90) | <0.001 |
| Healthy diet | 0.91 (0.89-0.94) | <0.001 |  | 0.93 (0.91-0.96) | <0.001 |
| Healthy sleeping | 0.72 (0.71-0.74) | <0.001 |  | 0.76 (0.74-0.78) | <0.001 |
| Moderate tea/coffee intake | 0.91 (0.88-0.94) | <0.001 |  | 0.93 (0.90-0.96) | 0.001 |
| No. of healthy lifestyle behaviors |  |  |  |  |  |
| 0-1 | 1(reference) |  |  | 1(reference) |  |
| 2 | 0.91 (0.88-0.95) | <0.001 |  | 0.94 (0.90-0.98) | 0.002 |
| 3 | 0.78 (0.75-0.81) | <0.001 |  | 0.82 (0.79-0.86) | <0.001 |
| 4 | 0.68 (0.65-0.71) | <0.001 |  | 0.74 (0.71-0.77) | <0.001 |
| 5-7 | 0.56 (0.53-0.58) | <0.001 |  | 0.63 (0.60-0.66) | <0.001 |
| *P* _for trend_ | 0.86 (0.86-0.87) | <0.001 |  | 0.89 (0.88-0.90) | <0.001 |
