# Supplementary material for: Healthy lifestyle as a protective factor against the risk of gastroesophageal reflux disease: Prospective population cohort analysis
Source: PLoS One. 2026 Oct 15;21(10):e0360299. doi: 10.1371/journal.pone.0360299 (PMC13671543; doi:10.1371/journal.pone.0360299)
Supplement: Supplementary file 10 [file pone.0360299.s010.docx]

**Table S8. Associations between healthy lifestyle behaviors (favorable, intermediate, and unfavorable classification) and risk of GERD.**

Notes: GERD, gastroesophageal reflux disease; HR, hazard ratio; CI, confidence interval; BMI, body mass index. Healthy lifestyle behaviors were categorized as favorable (score: 5 to 7), intermediate (score: 3 to 4), and unfavorable (score: 0 to 2).

Model 1: adjusted for age, gender.

Model 2: adjusted for age, gender, Townsend deprivation index, ethnics, occupation, education, history of cancer, diabetes, hypertension, depression, anxiety, upper gastroenterology diseases.

|  | **Model 1** | |  | **Model 2** | |
| --- | --- | --- | --- | --- | --- |
| **Healthy lifestyle behaviors** | **HR (95% CI)** | ***P* value** |  | **HR (95% CI)** | ***P* value** |
| Unfavorable | 1(reference) |  |  | 1(reference) |  |
| Intermediate | 0.85 (0.82-0.87) | <0.001 |  | 0.89 (0.86-0.92) | <0.001 |
| Favorable | 0.66 (0.64-0.69) | <0.001 |  | 0.73 (0.71-0.76) | <0.001 |
