# Supplementary material for: Healthy lifestyle as a protective factor against the risk of gastroesophageal reflux disease: Prospective population cohort analysis
Source: PLoS One. 2026 Oct 15;21(10):e0360299. doi: 10.1371/journal.pone.0360299 (PMC13671543; doi:10.1371/journal.pone.0360299)
Supplement: Supplementary file 13 [file pone.0360299.s013.docx]

# **Table S11. UK Biobank Field IDs of Variables**

| **Category** | **Variable** | **UKB Field ID(s)** | **Notes** |
| --- | --- | --- | --- |
| Outcome | Gastro-oesophageal reflux disease (GERD) | 131585, 131584 | First occurrence of ICD-10 K21 |
| Lifestyle Factors | Smoking status | 20116 | Never / former / current |
| Lifestyle Factors | Body mass index (BMI) | 21001 | Measured BMI |
| Lifestyle Factors | Physical activity | 22032 | Moderate/vigorous activity |
| Lifestyle Factors | Sleep duration | 1160 | Hours of sleep |
| Lifestyle Factors | Insomnia/sleeplessness | 1200 / 1210 | Sleep problems |
| Lifestyle Factors | Snoring | 1210 | Included in sleep score |
| Lifestyle Factors | Napping frequency | 1190 / 1200 | Included in sleep score |
| Lifestyle Factors | Ease of getting up | 1170 | Included in sleep score |
| Lifestyle Factors | Alcohol drinking frequency | 1558 | Frequency |
| Lifestyle Factors | Tea intake | 1488 | Cups/day |
| Lifestyle Factors | Coffee intake | 1498 | Cups/day |
| Lifestyle Factors | Diet components (FFQ) | 1289–1389 | Vegetable, fruit, fish, meat, etc. |
| Demographics | Age at recruitment | 21003 | Years |
| Demographics | Sex | 31 | Male/Female |
| Demographics | Ethnic background | 21000 | Ethnicity |
| Demographics | Townsend deprivation index | 22189 | Area deprivation |
| Demographics | Education | 6138 | Highest qualification |
| Demographics | Occupation | 6142 | Employment status |
| Health History | Diabetes | 20002 (multiple codes) | Self-reported |
| Health History | Hypertension | 20002 (multiple codes) | Self-reported |
| Health History | Depression | 20002 (multiple codes) | Self-reported |
| Health History | Anxiety | 20002 (multiple codes) | Self-reported |
| Health History | Upper GI diseases | 20002 (multiple codes) | Self-reported |
